# Makrozytäre Anämie und Polychondritis: VEXAS-Syndrom

**DOI:** 10.1007/s00393-023-01318-5

**Published:** 2023-02-03

**Authors:** Markus Zeisbrich, Viktoria Schindler, Máté Krausz, Michele Proietti, Pavla Mrovecova, Reinhard E. Voll, Cornelia Glaser, Fabian Röther, Klaus Warnatz, Nils Venhoff

**Affiliations:** 1 Klinik für Rheumatologie und Klinische Immunologie, Vaskulitis-Zentrum Freiburg, Universitätsklinikum Freiburg, Medizinische Fakultät, Albert-Ludwigs-Universität Freiburg, Freiburg, Deutschland; 2 Klinik für Rheumatologie und Klinische Immunologie, Universitätsklinikum Freiburg, Hugstetter Str. 55, 79106 Freiburg, Deutschland; 3 Centrum für chronische Immundefizienz, Universitätsklinikum Freiburg, Medizinische Fakultät, Albert-Ludwigs-Universität Freiburg, Freiburg, Deutschland; 4 Biologische Fakultät, Albert-Ludwigs-Universität Freiburg, Freiburg, Deutschland; 5 Praxis für Rheumatologie in Donaueschingen, Sonnenhaldenstr. 2, 78166 Donaueschingen, Deutschland

**Keywords:** Erworbene Mutation, Angeborenes Immunsysten, Autoinflammation, Unklares Fieber, Somatic mutation, Innate immunity, Autoinflammation, Fever of unknown origin

## Abstract

Das VEXAS-Syndrom (Akronym für Vacuoles, E1 enzyme, X‑linked, Autoinflammatory, Somatic) wurde Ende 2020 erstmals beschrieben und verursacht durch eine erworbene Mutation auf dem X‑Chromosom ein autoinflammatorisches Syndrom vorwiegend bei Männern im höheren Lebensalter. Klinisch zeichnet sich das VEXAS-Syndrom durch ein Mischbild aus rheumatologischer Erkrankung mit separaten hämatologischen Pathologien aus, wobei besonders häufig eine Polychondritis und fast immer eine makrozytäre Anämie auftreten. Anhand dieser Kasuistik werden die diagnostischen Kernpunkte zur Erkennung des VEXAS-Syndroms demonstriert.

## Abstract (en)

An adult-onset autoinflammatory syndrome caused by somatic mutations in the *UBA1* gene on the X chromosome was first reported in 2020. This VEXAS syndrome (acronym for vacuoles, E1 enzyme, X‑linked, autoinflammatory, somatic) is characterized by an overlap of rheumatic inflammatory diseases with separate hematologic abnormalities. A substantial number of affected patients suffer from treatment refractory relapsing polychondritis and nearly always show signs of macrocytic anemia. This case report illustrates the diagnostic key points to recognizing patients with VEXAS syndrome.

## Anamnese

Ein 79-jähriger Mann, der außer einer Osteoporose keine internistischen Vorerkrankungen aufwies, entwickelte plötzlich Fieber, Nachtschweiß und Polyarthralgien; laborchemisch bestand eine akute Entzündungskonstellation (s. Abb. 3). Im Rahmen einer stationären Abklärung konnte keine infektiöse Ursache ermittelt werden, ein Tumorscreening, bestehend aus Endoskopie, Sonographie, Computertomographie und Knochenmarkbiopsie, ergab keine Diagnose. Die rheumatologische Labordiagnostik inklusive Rheumafaktor, ANA- und ANCA-Screening zeigte sich unauffällig. Unter dem Verdacht auf eine autoinflammatorische Erkrankung erfolgte eine intravenöse Glukokortikoidtherapie, die zu einer Besserung des klinischen Zustands führte.

Im Rahmen der anschließenden Glukokortikoiddosisreduktion trat jedoch bereits bei 40 mg Prednisolon/Tag p.o. erneut Fieber auf, sodass zusätzlich eine Basistherapie mit Methotrexat 17,5 mg s.c./Woche initiiert wurde. Da auch unter dieser Therapie die Serum-CRP-Werte dauerhaft erhöht waren und sich der Allgemeinzustand bei jedem Kortison-Reduktionsschritt sukzessiv verschlechterte, wurde die Prednisolon-Therapie dauerhaft mit 40–50 mg/Tag fortgeführt, ohne dass sich die Entzündungsparameter (u. a. CRP) darunter dauerhaft normalisierten oder der Patient anhaltend entfieberte.

Im Rahmen der stationären Versorgung eines thromboembolischen Ereignisses nach 3‑monatiger GC-Therapie wurde bei persistierender Inflammation und Fieber vom konsultierten Rheumatologen eine IL-1-blockierende Therapie mit Anakinra (100 mg/Tag s.c.) begonnen. Hierunter war der Patient erstmals mehrere Wochen fieberfrei, und Prednisolon konnte erstmals bis auf 10 mg täglich reduziert werden. Weitere 2 Monate später wurde der Patient erneut aufgrund von Sinterungsfrakturen an Brust- und Lendenwirbelkörpern zur Kyphoplastie stationär aufgenommen. Aufgrund ausgeprägter Lokalreaktionen unter der Anakinra-Therapie, wurde die Therapie auf den IL-6-Rezeptorblocker Tocilizumab (162 mg s.c./Woche) umgestellt.

Auch unter Tocilizumab normalisierte sich das CRP nicht. Aufgrund einer neu aufgetretenen Leukopenie wurde das MTX-Applikationsintervall auf 14 Tage gestreckt, was einen erneuten entzündlichen Schub zur Folge hatte. Während dieses Schubes wurden nun auch Lokalreaktionen auf die subkutan applizierten Medikamente MTX und Tocilizumab beobachtet, sodass beide Medikamente abgesetzt und auf eine Behandlung mit dem JAK-Inhibitor Upadacitinib 15 mg/Tag umgestellt wurde. Da sich auch unter dieser Therapie ein refraktärer Verlauf mit dauerhaftem Steroidbedarf zeigte, erfolgte die stationäre Aufnahme in unserer Abteilung.

## Befund

Der Patient präsentierte sich zu diesem Zeitpunkt in deutlich reduziertem Allgemeinzustand, mit ungewolltem Gewichtsverlust von 10 kg innerhalb der letzten 12 Monate, täglich wiederkehrendem Fieber ≥ 38,5 °C, Nachtschweiß und Polyarthralgien. Hinweise auf eine kranielle oder extrakranielle Riesenzellarteriitis fanden sich bei der körperlichen Untersuchung nicht. Es fiel jedoch eine Druckdolenz des Nasen- und Ohrknorpels auf. Auf Nachfrage wurde ein Bestehen dieser Symptomatik seit etwa 2 Wochen angegeben. Unter der aktuellen Therapie mit Upadacitinib und Prednisolon (5 mg/Tag) bestanden Zeichen einer Akut-Phase-Reaktion mit erhöhtem Serum-CRP von 150 mg/l (Norm < 5 mg/l), Hyperferritinämie von 1965 ng/ml (Norm < 400 ng/ml) bei unwesentlich erhöhtem Procalcitonin von 0,12 ng/ml. Blutkulturen und Echokardiographie ergaben keine Hinweise auf eine Bakteriämie oder Endokarditis. Es zeigten sich eine hyperchrome, makrozytäre Anämie (MCV 103 fl) mit einem Hb-Wert von 8,7 g/dl sowie eine leichte Thrombozytose von 400–500 Tsd/µl. Bis auf eine monoklonale Gammopathie vom Typ IgG *kappa* war die immunologische Diagnostik inklusive breitem Autoantikörperscreening und Komplementdiagnostik unauffällig.

## Diagnose

### Weiteres Procedere und Diagnose Polychondritis

**Figure 1:**
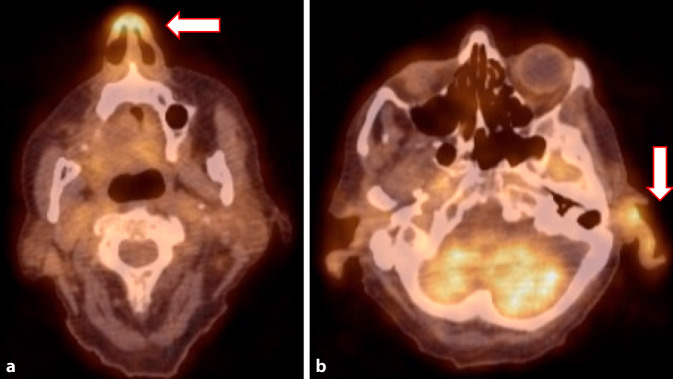

Aufgrund der therapierefraktären dauerhaften Inflammation erfolgte am Folgetag eine Ganzkörper-FDG-PET-CT zur Fokussuche. Passend zum klinischen Befund zeigte sich ein gesteigerter Glukosestoffwechsel der Ohrmuscheln beidseits sowie der knorpeligen Anteile des Nasenseptums (Abb. 1), außerdem vermehrte und vergrößerte zervikale Lymphknoten und eine Stoffwechselsteigerung des blutbildenden Knochenmarks. Hinweise auf eine entzündliche Beteiligung der Trachealknorpel und der Aorta ergaben sich nicht. Unter der Verdachtsdiagnose einer rezidivierenden Polychondritis leiteten wir eine Therapie mit dem TNF-α-Inhibitor Adalimumab (40 mg s.c. alle 14 Tage) ein, begleitend zu einer intravenösen Prednisolon-Therapie mit 60 mg/Tag. Bei deutlich gebessertem Allgemeinzustand und raschem CRP-Abfall auf 43 mg/l erfolgte die Entlassung mit ambulantem Steroidtapering.

### Stationäre Wiederaufnahme

**Figure 2:**
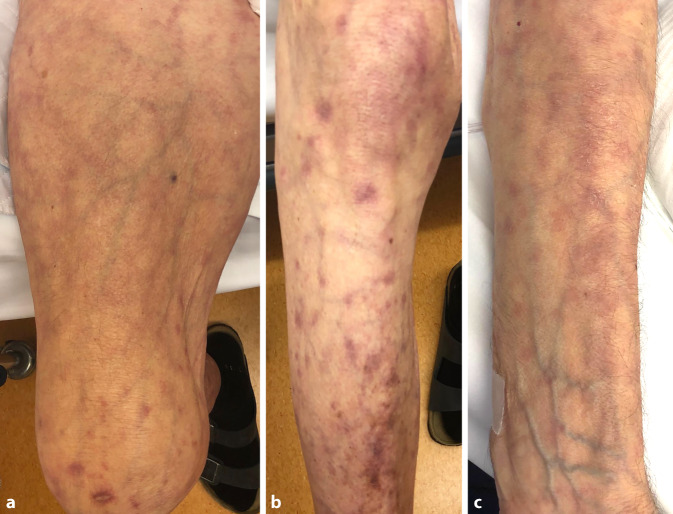

Nur wenige Wochen nach Entlassung wurde der Patient bei Verschlechterung des Allgemeinzustandes und Anstieg der Entzündungsparameter (CRP 180 mg/l) im Rahmen der Steroidreduktion erneut stationär aufgenommen. Bei insgesamt atrophem Hautbild zeigten sich eine Livedo racemosa an Armen und Beinen sowie teils purpuriforme und postinflammatorische Hautveränderungen (Abb. 2). Im CT des Thorax wurden erstmals pulmonale Infiltrate nachgewiesen.

Bei der kritischen Reevaluation aller Befunde und Neubewertung insbesondere der hyperchromen, makrozytären Anämie, die auch nach Pausieren der MTX-Therapie persistierte, zogen wir das Vorliegen des erst kürzlich beschriebenen VEXAS-Syndroms in Betracht, was in der nachfolgenden humangenetischen Diagnostik (Mutation c.122 T > C, p.Met41Thr hemizygot) bestätigt werden konnte.

### Definition

Die Erstbeschreibung des VEXAS-Syndroms (Akronym für Vacuoles, E1 enzyme, X‑linked, Autoinflammatory, Somatic) erfolgte Ende 2020 anhand einer Kohorte von 25 Männern, die eine somatische/erworbene Mutation des *UBA1*-Gens auf dem X‑Chromosom aufwiesen und unter einem inflammatorischen Syndrom litten [1]. Es handelt sich dabei um eine Loss-of-function-Mutation, die zu einer Störung der intrazellulären Ubiquitinierung und daraus folgend v. a. zu Autoinflammation und Blutbildveränderungen führt. Von den im peripheren Blut zirkulierenden Immunzellen tragen nur Zellen des angeborenen Immunsystems die Mutation (Neutrophile, Monozyten) – nicht aber die T‑ und B‑Lymphozyten. Die Erkrankung tritt erst im höheren Lebensalter auf (medianes Alter 64 Jahre; [1]) und aufgrund der X‑chromosomalen Vererbung fast ausschließlich bei Männern. Die bisher einzigen bei Frauen beschriebenen Fälle traten bei Patientinnen mit einer X‑chromosomalen Monosomie auf [2–4].

Die Krankheit ist gekennzeichnet durch einen meist therapierefraktären und komplikationsreichen Verlauf. Die Autoinflammation manifestiert sich oft mit klinischen Symptomen, durch die die Diagnose- oder Klassifikationskriterien rheumatologischer und hämatologischer Erkrankungen erfüllt werden. Den Hauptanteil an rheumatologischen Erkrankungen macht dabei die Polychondritis aus (15 von 25 Patienten in der Erstbeschreibung), vereinzelt kann es auch zu einer Panarteriitis nodosa kommen (3 von 25 Patienten in der Erstbeschreibung). Bisher nur im Einzelfall ist eine Assoziation mit einer Biopsie-gesicherten Riesenzellarteriitis (RZA) berichtet [1]. In einem weiteren Fall wurde zusätzlich zu einer Polychondritis eine Kontrastmittelaufnahme der A. subclavia als Riesenzellarteriitis gewertet [5]. Der tatsächliche Zusammenhang beider Erkrankungen bedarf weiterer Überprüfung, insbesondere da erste Screenings von englischen und französischen RZA-Kohorten negativ für Mutationen in *UBA1* ausfielen [6, 7].

Während sich die Hautläsionen von VEXAS-Patienten histopathologisch häufig als leukozytoklastische Vaskulitis klassifizieren lassen, wurde das Auftreten von anderen Kleingefäßvaskulitiden nur im Einzelfall mit unklarem tatsächlichem Zusammenhang berichtet [8].

Parallel können sich ein Sweet-Syndrom (8 von 25 Patienten in der Erstbeschreibung) oder hämatologische Erkrankungen wie ein myelodysplastisches Syndrom (MDS; 6 von 25 Patienten) oder ein Myelom bzw. eine monoklonale Gammopathie unklarer Signifikanz (5 von 25 Patienten) manifestieren. Nahezu alle Patienten weisen eine makrozytäre Anämie und im Knochenmarkbiopsat Vakuolen in myeloiden und erythrozytären Vorläuferzellen auf [1].

Die Diagnose erfolgt durch den Mutationsnachweis in myeloischen Zellen. Die Patienten benötigen fast immer hohe Glukokortikoiddosen, Einzelfallberichte über therapeutische Erfolge beinhalten die Blockade proinflammatorischer Zytokine wie IL‑1 und IL‑6 oder des JAK-Signalwegs, wobei Ruxolitinib gemäß erster Daten etwas besser als andere JAK-Inhibitoren zu wirken scheint [9]. Bei Vorliegen eines MDS existieren positive Berichte über den Einsatz von Azacitidin [10, 11], auch allogene Stammzelltransplantation wurde bereits erfolgreich durchgeführt [12, 13].

## Therapie und Verlauf

**Figure 3:**
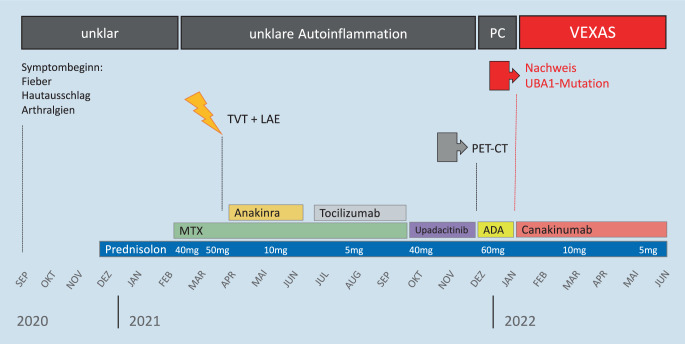

Aufgrund der Tatsache, dass in der Vergangenheit bei dem Patienten die IL-1-Blockade mit Anakinra als einzige Therapie zu Fieberfreiheit geführt hatte, begannen wir eine Therapie mit dem gegen IL-1β gerichteten monoklonalen Antikörper Canakinumab (150 mg s.c./alle 4 Wochen). Zum Zeitpunkt der stationären Entlassung betrug das CRP 87 mg/l nach durchgeführter Steroidstoßtherapie und erfolgter Erstgabe von Canakinumab. Innerhalb von 6 Monaten kam es unter langsamer Steroidreduktion und fortgeführter Canakinumab-Therapie zu einer allmählichen Stabilisierung des Allgemeinzustandes, erneute stationäre Aufenthalte waren nicht nötig. Im Rahmen des letzten Ambulanzbesuchs wurde bei einer täglichen Prednisolon-Dosis von 5 mg erstmals ein normwertiges CRP (< 5 mg/l) gemessen (Abb. 3).

## Diskussion

Dieser Fall verdeutlicht die Vielgestaltigkeit des VEXAS-Syndroms. Die unspezifischen, allgemeinen Symptome des Patienten begannen noch vor der Erstbeschreibung dieses autoinflammatorischen Syndroms, sodass die initial extern durchgeführte Knochenmarkpunktion entweder nicht hinsichtlich einer Vakuolenbildung analysiert wurde oder diese nicht aufgefallen ist. Mit dem heutigen Wissen über das VEXAS-Syndrom hätte die Diagnosefindung damals durch den Knochenmarkpunktionsbefund beschleunigt werden können.

Stattdessen kam es bei dem Patienten zu einem komplikationsreichen Krankheitsverlauf mit steroidbedingten Nebenwirkungen (Sinterungsfrakturen) und Erfüllung nahezu aller bisher beschriebenen Komplikationen im Rahmen des VEXAS-Syndroms (thromboembolisches Ereignis, pulmonale Infiltrate, Polychondritis, MGUS, Hautbeteiligung). Auch die bei unserem Patienten beobachteten Lokalreaktionen auf die subkutane Gabe von Anakinra wurden in der Erstbeschreibung des VEXAS-Syndroms bereits berichtet.

Besonders typisch ist das Auftreten einer Polychondritis in Zusammenhang mit einem VEXAS-Syndrom. Ferrada et al. [14] haben in einer Kohorte von 92 Patient*innen mit Polychondritis bei 7,6 % eine *UBA1*-Mutation nachweisen können, betrachtet man nur die männlichen Patienten, waren es sogar 50 %. Charakteristisch für die Polychondritis bei VEXAS-Patienten war die fehlende Beteiligung der unteren Atemwege, ein Befund, der auch auf den in dieser Kasuistik vorgestellten Patienten zutrifft. Typisch für eine Polychondritis bei zugrunde liegendem VEXAS-Syndrom war in der genannten Kohorte außerdem eine Thrombopenie – dieser Befund trifft auf den hier vorgestellten Patienten nicht zu, bei dem nur normwertige oder erhöhte Thrombozytenwerte dokumentiert sind.

Die Herausforderung der Diagnosefindung bei VEXAS-Patienten besteht darin, dass sie mitunter die Diagnosekriterien von rheumatologischen und hämatologischen Krankheitsbildern erfüllen können und so möglicherweise falsch oder nur unzureichend behandelt werden. Auch wir haben zunächst eine Polychondritis diagnostiziert und die MGUS und die Anämie als Begleiterkrankungen bei einem älteren Patienten interpretiert. Erst der weiterhin therapierefraktäre Verlauf und v. a. die Neubewertung der makrozytären Anämie bei einem älteren männlichen Patienten führten in Kenntnis der aktuellen Literatur zur genetischen Sicherung der korrekten Diagnose eines VEXAS-Syndroms.

## Fazit für die Praxis

- Das VEXAS-Syndrom ist autoinflammatorisches Krankheitsbild mit hohem Steroidbedarf und häufig therapierefraktärem Verlauf bei vorwiegend älteren männlichen Patienten.
- Relativ oft treten eine Polychondritis und begleitende Hautveränderungen auf. Fast immer besteht eine makrozytäre Anämie, aber auch andere hämatologische Veränderungen wie eine MGUS oder ein MDS treten regelmäßig auf.
- Die Diagnose erfolgt durch den Mutationsnachweis in myeloischen Zellen und den Nachweis von Vakuolen in myeloiden und erythrozytären Vorläuferzellen im Knochenmark.
